# The Development Strategy of Home-Based Exercise in China Based on the SWOT-AHP Model

**DOI:** 10.3390/ijerph18031224

**Published:** 2021-01-29

**Authors:** Hanming Li, Xingquan Chen, Yiwei Fang

**Affiliations:** 1 College of Physical Education, Sichuan University, Chengdu 610065, China; lhm@stu.scu.edu.cn; 2 Department of Materials Science and Chemical Engineering, Stony Brook University, Stony Brook, NY 11794, USA; yiwei.fang@stonybrook.edu

**Keywords:** home-based exercise, sustainable development, SWOT-AHP, physical activities, public health, COVID-19, intelligent sports

## Abstract

In view of the increasing importance of sports to people and the impact of COVID-19 on people’s lives, home-based exercise has become a popular choice for people to keep fit due to its unique advantages and its popularity is expected to keep growing in the future. Therefore, it is necessary to determine the development direction of home-based exercise and put in the corresponding efforts. However, there is currently a lack of research on all aspects of home-based exercise. The purpose of this research was to investigate the effective sustainable development strategy of home-based exercise in China through a SWOT (Strengths, Weaknesses, Opportunities and Threats) and AHP (Analytic Hierarchy Process) hybrid model. Thirteen factors corresponding to the SWOT analysis were identified through a literature review and expert opinions. The results show that in China the advantages and potential outweigh the weaknesses and threats of home-based exercise. Home-based exercise should grasp the external development opportunities and choose the SO development strategic type that combines internal strengths and external opportunities. As the core for the development of home-based exercise, this strategy should be given priority. To sum up, home-based exercise is believed to have a bright future.

## 1. Introduction

At the end of 2019, the COVID-19 epidemic broke out and spread all over the world. During the prevention and control period of the pandemic, various sports venues were not open. This scenario where all sports venues are shut down is challenging to everyone who needs exercise. In January 2020, the General Administration of Sports of China issued the “Notice on Vigorously Promoting Scientific Home-based Exercise Methods”, requesting local sports departments to introduce simple, scientific, and effective home-based exercise methods based on local conditions. Since then, “home-based exercise” has gradually become a hot topic in China.

The idea of home-based exercise is to fully utilize the space in your home and make it act as a sports venue, with your own or family members, with your hands or using some portable equipment, to perform a series of applicable sports to strengthen your physical fitness and mentality. Home-based exercise is popular among people of all ages due to its convenience and is one of the important ways to stay healthy. People carry out various forms of sports at home, which not only strengthen their physical fitness and improve their own immunity, but also help relieve a series of psychological problems caused by COVID-19 [1,2,3,4].

Before the outbreak of the epidemic, home-based exercise was mainly used as aids for patient treatment or as means to restore physical function after surgery. Existing researches show that home-based exercise has good auxiliary effects on the treatment of fractures, osteoarthritis, and other musculoskeletal system diseases [5,6,7,8,9,10], cardio-cerebral vascular system diseases [11,12,13,14,15,16], respiratory system diseases [17,18,19], and even cancers [20,21,22,23]. Home-based exercise also has effects on supporting the treatment of nervous system diseases such as depression and Parkinson’s disease [24,25,26,27,28].

After the outbreak of the epidemic, in order to reduce people going out and avoid too much contact with each other to cause infection, home-based exercise replaced sports outdoors or in specific venues and became an inevitable choice to meet people’s needs. According to the study of Bo Pu et al. [29], during the epidemic, people mainly carried out the five aspects of home-based exercise, that is, gymnastics, walking or jogging, stretching exercises, housework, etc. It shows that male, married, and more than 25-year-old participants are more likely to do home-based exercise [29,30,31,32].

As COVID-19 still poses a huge threat to the public health, more and more people around the world have adopted home-based exercise. Home-based exercise has the prospect of becoming a common way for people to participate in physical activities. This study will employ a SWOT (Strengths, Weaknesses, Opportunities and Threats) and AHP (Analytic Hierarchy Process) hybrid model to provide a certain reference for the sustainable development of home-based exercise in China, and also it can provide some references for the development of home-based exercise in other countries.

### 1.1. SWOT Analysis

The SWOT analysis is a widely used tool for analyzing internal and external environments in order to attain a systematic approach and support for decision situations [33]. By applying the SWOT analysis in scientific research, people are able to draw a series of conclusions from the research objects including the main strengths, weaknesses, opportunities, and threats. Most articles on the SWOT analysis only presented a literal description of the analysis and a few conducted quantified analysis and as planning processes are often complicated by numerous criteria and interdependencies, it may lead to the insufficient use of this analytical method [33,34,35].

### 1.2. Analytic Hierarchy Process (AHP)

The analytical hierarchy process (AHP) is a multiple criteria decision analysis (MCDA) method which helps in addressing the complicated decision problems [36,37]. The advantages of AHP include its ability to qualitatively and quantitatively analyze decision attributes, and its flexibility with regard to the setting of objectives [38]. It does so by structuring the problem, identifying decision making factors, measuring the importance of the factors, and synthesizing all the decision-making factors [39,40,41]. At present, AHP has been widely used in various areas, such as operations management [42], health care [43], project risk assessment [44], etc. [45,46]. However, AHP also has some defects, for example, it is criticized for its possible rank reversal phenomenon [47].

### 1.3. SWOT-AHP Model

Since the SWOT analysis includes no quantitative analysis, AHP can be integrated with the SWOT analysis [48,49,50]. Research by Mika Marttunen et al. shows that SWOT is most often used in combination with MCDA methods, and the mixed use of SWOT and AHP is the most common one [51]. Using AHP, each group of SWOT can be created as a pairwise comparison matrix, the weights and intensities of the SWOT groups and factors can be measured [50]. In this way, the reliability of the SWOT analysis can be improved. Many studies in different disciplines have already achieved good results by utilizing the SWOT-AHP hybrid model [38,48,52,53], and this method has also been applied in sports science and physical education [39,54,55,56].

## 2. Methods

### 2.1. Factors Generation

In order to determine the factors, the expert panel of sports science and medical science conducts a SWOT analysis. First, 10 expert panel members leading by Prof. Xingquan Chen from the Physical Education College of Sichuan University and West China Hospital of Sichuan University conducted the SWOT analysis. Every member of the panel was asked to identify various strengths and weaknesses, opportunities and threats related to the development of home-based exercise. Based on the SWOT analysis, the authors selected 13 factors. Then, these factors were grouped into each SWOT category and were given a brief description (Table 1).

### 2.2. AHP Instrument

**Figure 1:**
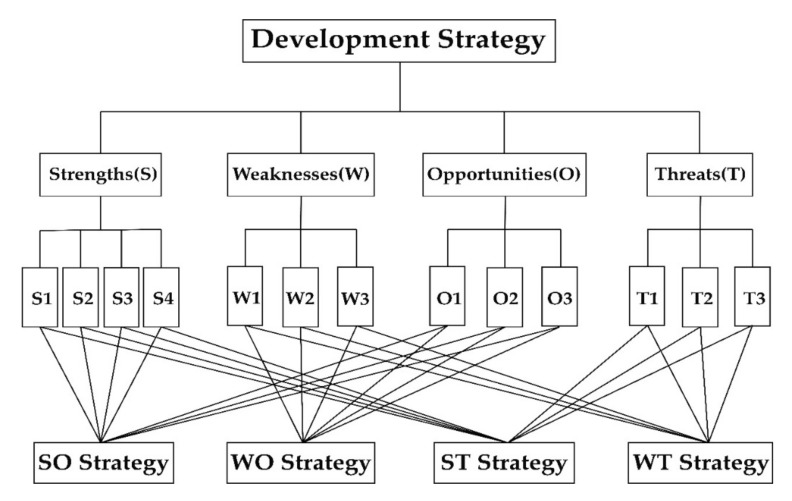
Hierarchical analysis structure graph.

Based on the factors obtained by the SWOT analysis method, the AHP hierarchy was constructed (Figure 1). In order to carry out the analytic hierarchy process, a questionnaire was compiled that required a series of paired comparisons of factors. The respondents were asked to give answers by comparing two given factors according to his or her own preferences. They were asked to evaluate the factors based on the AHP scale (Table 2) [40].

### 2.3. Weight and Consistency Check

Since people tend to make inconsistent decisions, decision making science should judge the consistency of decision making, a consistency ratio (CR) test is a measurement of the validity of the survey respondents’ responses [39,40,41]. IBM SPSS Statistics 23 and MATLAB R2018b were used for statistics and calculations. The calculation methods and steps are as follows [40,53,54]:

1. Construct comparison matrix A.

(1) A=aij=a11a12⋯a1na21a22⋯a2n⋮⋮⋱⋮an1an2⋯ann=1w1w2⋯w1wnw2w11⋯w2wn⋮⋮⋱⋮wnw1wnw2⋯1

2. Calculate the geometrical mean ($\bar{W_{i}}$) of each row of the judgment matrix using the product square root method.

(2) $$\bar{W_{i}}={\left(\overset{n}{\underset{j=1}{\prod }}a_{ij}\right)}^{\frac{1}{n}}i,j=1,2,\cdots ,n$$

3. Normalize the geometrical mean of each row to get the eigenvectors ($W_{i}$).

(3) $$W_{i}=\frac{\bar{W_{i}}}{\sum_{j=1}^{n}\bar{w_{j}}}\;i,j=1,2,\cdots ,n$$

4. Calculate the maximum eigenvalue ($\lambda_{max}$) of the judgment matrix.

(4) $$\lambda_{max}=\frac{1}{n}\overset{n}{\underset{i=1}{\sum }}\frac{\left(\sum_{j=1}^{n}a_{ij}W_{j}\right)}{W_{i}}\;i,j=1,2,\cdots ,n$$

5. Calculate the consistency index (CI) and the consistency ratio (CR).

(5) $$CI=\frac{\lambda_{max}-n}{n-1}$$

When n>2, CR represents the consistency of the matrix, RI values are shown in Table 3.

(6) $$CR=\frac{CI}{RI}$$

If  CR < 0.1, the judgment matrix of the index meets the requirements of the consistency test.

### 2.4. The Calculation of the Intensity of Factors and SWOT Strategic Quadrilateral

The magnitude of the factor’s effect is intensity, and its actual level is the estimated strength, then intensity=estimated strength×weight. The estimated strength of each factor is represented by 0–5 points, *S*, *O* are represented by positive values, *W*, *T* are represented by negative values, the greater the absolute value, the greater the intensity.

The four variables of *S*, *W*, *O*, and *T* total intensity are each semi-axis, forming a four semi-dimensional coordinate system. Draw the velocity values *S*’, *W*’, *O*’, and *T*’ on the corresponding semi-axes of the coordinate system to obtain a strategic quadrilateral.

### 2.5. The Calculation of the Strategic Vector $\left(\theta ,\rho \right)$

In the SWOT-AHP model, the strategic azimuth angle θ is used to judge the strategic type, and the strategic intensity coefficient ρ is used to judge the strategic intensity. In the polar coordinates of the strategic type and strategic intensity spectrum, the coordinates $\left(\theta ,\rho \right)$ form a strategic vector with θ as the azimuth angle and ρ as the polar diameter.

Calculate the strategic azimuth θ, the center of gravity coordinate is:

(7) $$P\left(X,Y\right)=P\left(\frac{\sum xi}{4},\frac{\sum yi}{4}\right)$$

The strategic azimuth is:

(8) $$\theta =arctan\frac{Y}{X}\;\left(0\leq \theta \leq \pi \right)$$

Among them, xi and yi are the coordinates of *S*’, *W*’, *O*’, *T*’ in the strategic quadrilateral, respectively.

Calculate the strategic strength coefficient ρ.

The strategic positive intensity is:

(9) $$U=O^{\prime }\times S^{\prime }$$

The strategic negative intensity is:

(10) $$V=T^{\prime }\times W^{\prime }$$

The strategic intensity coefficient is defined as:

(11) $$\rho =\frac{U}{U+V}$$

The value range of ρ is $\left[0,1\right]$, and the size of ρ indicates the intensity of the strategic type.

## 3. Results

### 3.1. AHP Weights and the Intensities of Factors

The results obtained in Table 4 and Table 5 show that opportunities are the most important consideration, followed by strengths, weaknesses, and threats. Under the strength category, construction of a leading sports nation was rated as the most important factor, followed by an increased awareness of exercise, time freedom, low cost, and convenience. Under weakness, less theoretical research and insufficient professional talents were rated as the most important factors, followed by limited space leads to fewer sports methods and monotonous and boring form of exercise. Under opportunity, the rapid development of intelligent sports was rated as the most important factor, followed by support provided by the government and the stable development of the sports industry. Under threat, fading enthusiasm for home-based exercise after the epidemic was rated as the most important factor, followed by easy to slack at home and noise.

### 3.2. SWOT Strategic Quadrilateral

**Figure 2:**
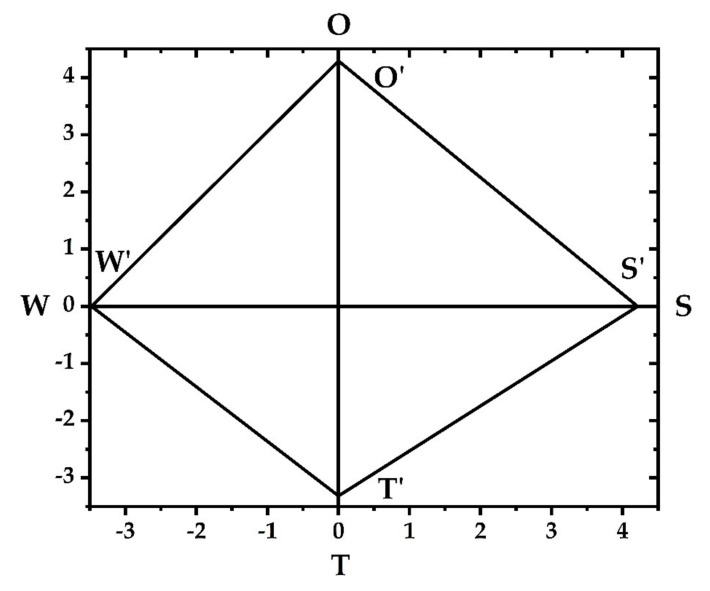
SWOT strategic quadrilateral.

The strategic quadrilateral (Figure 2) was drawn based on the calculation results of the total intensities of each group. The results are shown in Table 5:

(12) ∑Oi=4.2901>∑Si=4.2112>∑Wi=−3.4786>∑Ti=−3.3144.

### 3.3. Strategic Vector $\left(\theta ,\rho \right)$

The center of gravity coordinate is:$\;\left(0.1832,0.2439\right)$.
The strategic azimuth is: $\theta =arctan\left(\frac{0.2439}{0.1832}\right)\approx 53.09{}^{\circ}\left(0\leq \theta \leq \pi \right)$.
The strategic positive intensity is: U=18.0665
The strategic negative intensity is: V=11.5295
The strategic strength coefficient is: ρ=0.6104

**Figure 3:**
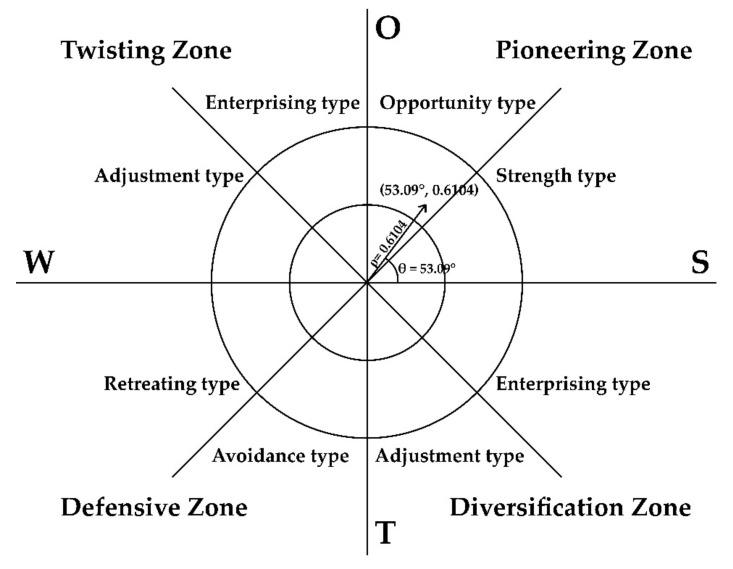
Strategic type and strategic intensity diagrams.

It can be seen from Figure 3 that the coordinates are $\left(\theta ,\rho \right)=\left(53.09{}^{\circ},0.6104\right)$, indicating that the development of home-based exercise has a greater opportunity and its inherent advantages are also obvious.

## 4. Discussion

The results of this study show that opportunities and strengths are more important than weaknesses and threats. It is concluded that home-based exercise in China should adopt the type of SO development strategy that combines internal advantages and external opportunities. When developing the home-based exercise with its featured advantages, its shortcomings should also be improved at the same time. The implementation focus of the development strategy of home-based exercise in China are as follows.

First, in the strength group, the construction of a leading sports nation is the highest rated factor, and under opportunity, support provided by the government has a high importance. With the support of various policies [57,58,59] and the stable development of China’s sports industry [67,68], the environment of sports in China will get better and better. It is necessary to seize this hard-won opportunity to improve the development of home-based exercise. Home-based exercise should be further promoted to the public with its unique strengths and it will become more popular in the future.

Second, in the opportunity group, the rapid development of intelligent sports is the highest rated factor. With the development of economy and technology in recent years, intelligent sports facilities are also constantly updated [62,63,64,65,66,69,70,71]. During the COVID-19 epidemic, various intelligent sports facilities also effectively encouraged people’s physical activities [72]. In the future, the home will become an important scene for the use of sports equipment. Home-based exercise will advance the development of intelligent sports, and the rapid development of intelligent sports can also propagate the home-based exercise development.

Third, in the weakness group, less theoretical research and insufficient professional talents have a relatively high importance. Home-based exercise hardly attracted attention in the past and few people have conducted targeted research on it. It is still used as a means of rehabilitation after illness or after surgery, rather than as general exercise methods for comprehensive analysis and research [73]. However, during the epidemic, more and more people realized the necessity of home-base exercise and used it as a daily approach to keep healthy and improve mental well-being [74]. Therefore, scholars and researchers should be encouraged to actively carry out empirical research on the home-based exercise, enrich and improve the theoretical system, and fill in academic gaps.

Fourth, under threat, fading enthusiasm for home-based exercise after the epidemic was the highest rated factor. The rapid rise of home-based exercise is mainly due to the epidemic which prevents people from participating in outdoor sports [2,72,74,75]. However, at present, the epidemic prevention and control situation in China is promising, and sports venues are gradually opening up, resulting in a decrease in the number of home-based exercisers. If home-based exercise and outdoor sports are combined and developed in coordination, a new pattern of sports for all will form.

Finally, the lower rated factors in weakness and threat groups also cannot be ignored. As mobile sports apps become more practical, the number of people studying online sports courses is also increasing [76,77,78]. Traditional online sports courses are mainly based on physical exercise such as muscle strength and flexibility training, which is pretty boring for people. Changes in the form of courses can be made in order to increase the joy of taking courses, which can increase people’s enthusiasm for participating in home-based exercise.

## 5. Conclusions

The COVID-19 epidemic has a great impact on all walks of life, but for home-based exercise, it is a golden opportunity for development. This article uses the SWOT-AHP hybrid model to conduct empirical research on the development of home-based exercise in China. The results indicate that strengths and opportunities have a greater influence on the development of home-based exercise than weaknesses and threats in the four dimensions of SWOT analysis. With the support of the various policies issued by governments and the rapid development of intelligent sports, home-based exercise should grasp the external development opportunities and choose the SO development strategic type that combines internal advantages and external opportunities. However, home-based exercise still has disadvantages such as limited space, low interest, and potential disturbance to the people. However, these problems can be solved with the development of science and technology. Home-based exercise will eventually form a culture and be thoroughly integrated into the lives of people.

The limitation of this study is the limited number of experts participating in the SWOT analysis and the limited SWOT factors, and the conclusions obtained may have deviations within an acceptable range. Future research will involve more experts in different fields for further analysis. The opinions of experts in different fields can be compared. The SWOT factors will be expanded, and the SWOT analysis can be combined with different MCDA methods for a comparative analysis to obtain more reliable data. Due to the bright future of intelligent sports facilities, research on their application in different sports will also be conducted in the future.

## Figures and Tables

**Table 1:** Strengths, weaknesses, opportunities and threats-analytical hierarchy process (SWOT-AHP) factors and description.

SWOT Group	SWOT Factor	Description of Factor
Strengths (S)	S1 Construction of a leading sports nation	The issuance of various policies has promoted China’s sports industry [57,58].
	S2 Increased awareness of exercise	People have gradually realized the importance of physical health.
	S3 Time Freedom	Home-based exercise can meet the demand of most office workers to do sports.
	S4 Low cost and convenient	People can complete the sports with light equipment or just with hands, and home-based exercise is not affected by the weather and is more convenient.
Weaknesses (W)	W1 Limited space leads to fewer sports methods	The equipment that can be used is basically simple and lightweight and there are fewer options to do sports at home, which are more restrictive.
	W2 Monotonous and boring form of exercise	Home-based exercise is less interesting and easy to cause boredom.
	W3 Less theoretical research and insufficient professional talents	There are relatively few theoretical studies and there is still a lack of innovative talents who can cross-study sports with other disciplines.
Opportunities (O)	O1 Support provided by the government	“Notice on Vigorously Promoting Scientific Home-based Exercise Methods” issued by the General Administration of Sports of China provides strong support for the future development of home-based exercise [59].
	O2 The stable development of sports industry	The output value of China’s sports industry and the scale of the online sports market have grown steadily every year, laying a solid foundation for the development of home-based exercise [60,61].
	O3 The rapid development of intelligent sports	Wearable devices, intelligent sports equipment, intelligent sports entertainment products, virtual reality technology, artificial intelligence motion algorithms and various new environmentally friendly materials used in sports equipment are also key projects for the development of the intelligent sports industry [62,63,64,65,66].
Threats (T)	T1 Noise	It is very likely that excessive noise will be produced, affecting neighbors.
	T2 Easy to be slack at home	Exercise at home can easily make people slack and become lazy.
	T3 Fading enthusiasm for home-based exercise	After the epidemic, the enthusiasm for home exercise easily fades.

**Table 2:** The fundamental scale of absolute numbers.

Intensity of Importance	Definition	Explanation
1	Equal Importance	Two activities contribute equally to the objective
3	Moderate importance	Experience and judgement slightly favor one activity over another
5	Strong importance	Experience and judgement strongly favor one activity over another
7	Very strong or demonstrated importance	An activity is favored very strongly over another; its dominance is demonstrated in practice
9	Extreme importance	The evidence favoring one activity over another is of the highest possible order of affirmation
2,4,6,8	Importance between the above levels	
Reciprocals of the above	If activity i has one of the above non-zero numbers assigned to it when compared with activity j, then j has the reciprocal value when compared with i	A reasonable assumption

**Table 3:** Average random consistency index.

n	1	2	3	4	5	6	7	8	9	10
RI	0	0	0.58	0.90	1.12	1.24	1.32	1.41	1.45	1.49

**Table 4:** Comparison matrix and weights of SWOT groups and factors.

SWOT Group	Comparison Matrix	Factor Weight	$\mathrm{Maximum}\;\mathrm{Eigenvalue}\;(\lambda_{max})$	Consistency Index (CI)	Consistency Ratio (CR)
Strengths (S)	$\left[\begin{array}{cccc} 1 & 2 & 4 & 5\\ 1/2 & 1 & 3 & 4\\ 1/4 & 1/3 & 1 & 2\\ 1/5 & 1/4 & 1/2 & 1 \end{array}\right]$	WS1 = 0.4915	4.0484	0.0161	0.0179
		WS2 = 0.3059			
		WS3 = 0.1249			
		WS4 = 0.0777			
Weaknesses (W)	$\left[\begin{array}{ccc} 1 & 2 & 1/4\\ 1/2 & 1 & 1/3\\ 4 & 3 & 1 \end{array}\right]$	WW1 = 0.2184	3.1078	0.0539	0.0929
		WW2 = 0.1515			
		WW3 = 0.6301			
Opportunities (O)	$\left[\begin{array}{ccc} 1 & 3 & 1/4\\ 1/3 & 1 & 1/6\\ 4 & 6 & 1 \end{array}\right]$	WO1 = 0.2176	3.0536	0.0268	0.0462
		WO2 = 0.0914			
		WO3 = 0.6909			
Threats (T)	$\left[\begin{array}{ccc} 1 & 1/3 & 1/4\\ 3 & 1 & 1/2\\ 4 & 2 & 1 \end{array}\right]$	WT1 = 0.1220	3.0183	0.00915	0.0158
		WT2 = 0.3196			
		WT3 = 0.5584			

CR<0.1, pass the consistency check.

**Table 5:** The intensities of groups and factors.

SWOT Group	Factor Weight	Estimated Strength	Factor Intensity	Total Intensity
Strengths (S)	W_S1_ = 0.4915	5	2.4575	∑Si=4.2112
	W_S2_ = 0.3059	4	1.2236	
	W_S3_ = 0.1249	3	0.3747	
	W_S4_ = 0.0777	2	0.1554	
Weaknesses (W)	W_W1_ = 0.2184	−3	−0.6552	∑Wi=−3.4786
	W_W2_ = 0.1515	−2	−0.3030	
	W_W3_ = 0.6301	−4	−2.5204	
Opportunities (O)	W_O1_ = 0.2176	3	0.6528	∑Oi=4.2901
	W_O2_ = 0.0914	2	0.1828	
	W_O3_ = 0.6909	5	3.4545	
Threats (T)	W_T1_ = 0.1220	−1	−0.1220	∑Ti=−3.3144
	W_T2_ = 0.3196	−3	−0.9588	
	W_T3_ = 0.5584	−4	−2.2336	
